# Impact of *KCNJ11*, *TCF7L2*, *SLC30A8*, *IGF2BP2*, *PPARG*, *SLC47A1*, *STK11*, *HHEX*, *KCNQ1*, *CDKAL1*, *FTO*, *CYP2C9*, *ADIPOQ*, *CAPN10* gene polymorphisms on risk of type 2 diabetes and therapeutic response to sulfonylurea and metformin therapy

**DOI:** 10.1186/1755-8166-7-S1-P100

**Published:** 2014-01-21

**Authors:** Nagaraja Phani, Padmalatha Rai, Prabha Adhikari, Shivashankara Nagri, Sydney D’Souza, Mundyat Gopinath, Kapaettu Satyamoorthy

**Affiliations:** 1 Division of Biotechnology, School of Life Sciences, Manipal University, Manipal, India; 2 Department of Medicine, Kasturba Medical College, Manipal University, Mangalore, India; 3 Department of Medicine, Kasturba Medical College, Manipal University, Manipal, India

## 

Type 2 Diabetes (T2D) represents a spectrum of metabolic disorders is genetically heterogeneous disease with multiple genes located on different chromosomes contributing to its susceptibility. Prevalence of T2D has increased sharply in recent years with more than 300 million people worldwide and 70 million in India. The application of molecular, genomic knowledge will provide new opportunities to explore the heterogeneity which patients clearly exhibit and provide a more accurate understanding of individual patients. Single nucleotide polymorphisms (SNPs) represent the most profuse form of genetic variation in humans which are gaining increased popularity and emerging as a new generation genetic markers. With several classes of drugs available to treat T2D its clinical response exhibits significant variation among individuals which exerts a huge impact on healthcare system and economic burden due to diabetes treatment. Pharmacogenetic research which assesses the role of genetic variants can be used in elucidating the nature of these variants on differential drug response. In the current study, we evaluated the association of *KCNJ11*, *TCF7L2*, *SLC30A8*, *IGF2BP2*, *PPARG*, *SLC47A1*, *STK11*, *HHEX*, *KCNQ1*, *CDKAL1*, *FTO*, *CYP2C9*, *ADIPOQ*, *CAPN10* gene polymorphisms with T2D and response to sulfonylurea and metformin using PCR-RFLP, TETRA-ARMS and DNA sequencing techniques. Study subjects were 330 T2D patients who are on sulfonylurea (178) and metformin (152) treatment with age and sex matched normal healthy controls of south Indian origin. Allele frequencies, genotype and haplotype distribution were analyzed by chi-squared test. Logistic regression analysis was used to predict the effect of gene variants on treatment. Gene–gene interactions were analyzed by generalized multifactor dimensionality reduction method. Linkage disequilibrium between each pair of SNP loci, were estimated and plotted with JLIN. We have also performed a systematic review and Meta-Analysis of KCNJ11 rs5219 and PPARG rs1801282 SNPs on South Asian populations to clarify inconsistency in association results. Our analysis showed KCNJ11 rs5215, SLC30A8 rs1326634, *TCF7L2* rs7903146 were associated with susceptibility to T2D. Multivariate regression analysis showed that *TCF7L2* rs12243326 TT genotype, KCNJ11 rs5219 TT genotype were associated with response rate to sulfonylurea treatment and STK11 rs741765 GG genotype, SLC30A8 rs1326634 TT genotype were associated with response rate to metformin treatment.

